# A Systematic Comparison of Age and Gender Prediction on IMU Sensor-Based Gait Traces

**DOI:** 10.3390/s19132945

**Published:** 2019-07-04

**Authors:** Tim Van hamme, Giuseppe Garofalo, Enrique Argones Rúa, Davy Preuveneers, Wouter Joosen

**Affiliations:** 1 imec-DistriNet, KU Leuven, Celestijnenlaan 200A, B-3001 Heverlee, Belgium; 2 imec-COSIC, KU Leuven, Kasteelpark Arenberg 10, B-3001 Heverlee, Belgium

**Keywords:** gait, age, gender, accelerometer, prediction

## Abstract

Sensors provide the foundation of many smart applications and cyber–physical systems by measuring and processing information upon which applications can make intelligent decisions or inform their users. Inertial measurement unit (IMU) sensors—and accelerometers and gyroscopes in particular—are readily available on contemporary smartphones and wearable devices. They have been widely adopted in the area of activity recognition, with fall detection and step counting applications being prominent examples in this field. However, these sensors may also incidentally reveal sensitive information in a way that is not easily envisioned upfront by developers. Far worse, the leakage of sensitive information to third parties, such as recommender systems or targeted advertising applications, may cause privacy concerns for unsuspecting end-users. In this paper, we explore the elicitation of age and gender information from gait traces obtained from IMU sensors, and systematically compare different feature engineering and machine learning algorithms, including both traditional and deep learning methods. We describe in detail the prediction methods that our team used in the OU-ISIR Wearable Sensor-based Gait Challenge: Age and Gender (GAG 2019) at the 12th IAPR International Conference on Biometrics. In these two competitions, our team obtained the best solutions amongst all international participants, and this for both the age and gender predictions. Our research shows that it is feasible to predict age and gender with a reasonable accuracy on gait traces of just a few seconds. Furthermore, it illustrates the need to put in place adequate measures in order to mitigate unintended information leakage by abusing sensors as an unanticipated side channel for sensitive information or private traits.

## 1. Introduction

Inertial measurement units (IMU) are packages that combine multi-axis acceleration and gyroscope sensors to measure motion or the direction of movement [1]. They are frequently used in advanced consumer electronics, such as wearable devices and smartphones, for games [2] and healthcare applications [3,4,5], but also in robots for industrial applications [6,7]. Contrary to environmental sensors, the output of acceleration and gyroscope sensors often requires calibration and further signal processing to elicit relevant features to automate decisions or inform their users.

The scope of this work is the use of IMU sensors for gait analysis [8,9,10,11]. Beyond the typical step counting and fall detection applications, gait information can offer good indicators to recognize certain health conditions, such as Parkinson’s disease [12,13]. In the area of security, the 3D acceleration and angular velocity in someone’s gait can be used as a soft biometric to authenticate someone by the way the walk [14,15,16]. Indeed, accelerometers and gyroscopes can offer a wealth of information to make applications smart. However, while the initial intent of applications using IMU sensors might be benign, the sensors may leak sensitive information about the subject in a way that is not easily understood or anticipated upfront.

In this paper, we study the feasibility of exfiltrating age and gender information from gait traces of only a few seconds long. This study was carried out in the frame of the OU-ISIR Wearable Sensor-based Gait Challenge: Age and Gender (GAG 2019) competition at the 12th IAPR International Conference on Biometrics (http://www.am.sanken.osaka-u.ac.jp/GAG2019/). We describe and compare the different traditional machine learning and deep learning techniques our team used to predict both age and gender information. In both the age and gender competitions, our team obtained the best solutions amongst all international participants, as confirmed by the competition organizers in  [17]. Our research shows that it is feasible to predict age and gender with a reasonable accuracy on short gait traces. Furthermore, it illustrates the need to put in place adequate measures in order to mitigate unintended information leakage, such that sensors cannot be abused as a side channel that reveals sensitive information or private traits.

In Section 2, we describe relevant related work on gait analysis and the elicitation of age and gender information. Section 3 elaborates on the gait dataset, the pre-processing methods and the experimental protocol. The systematic comparison was carried out according to this experimental protocol for the machine learning pipelines described in Section 4. Section 5 reports about the outcome of our evaluation. We conclude in Section 6 summarizing our main insights and directions for further research.

## 2. Related Work

The field of activity recognition using accelerometer sensors has produced a fairly large body of knowledge. Discussing these scientific results is beyond the scope of the work. We will highlight some examples on the use of IMU sensors for gait analysis, before we continue more specifically how gait traces are used to elicit age and gender information. For a more elaborate overview on gait recognition, we refer to relevant surveys [11,18,19].

### 2.1. Gait Analysis

Anwary et al. [20] investigated the optimal location and orientation of placing an IMU sensor on the barefoot and the parameters that influence the automatic extraction of gait features for estimating the distance during normal walking on ground level. The method they proposed aims to analyze the accelerometer and gyroscope data for the number of strides, distance, speed, length and period of stride, stance, and swing phases during walking. The data was gathered by placing sensors in five selected foot locations, and the best results were obtained for the sensor located at the centre point of foot. The authors were able to achieve an accuracy on the detection of the numbers of strides of about 95.47% using the accelerometer and about 93.60% with the gyroscope. Similar to other researchers, their experiments demonstrated that the sensor orientation and small changes of sensor location can influence the sensor output.

Qiu et al. [21] specifically investigated how human lower limbs move based on the Denavit–Hartenberg (D-H) convention. They used distributed wearable sensors augmented with domain-specific assumptions and the kinematic chain when the foot stays stationary on the ground, which imposes constraints that help minimize the estimation error of the knee position. More specifically, they used three wearable magnetic angular rate and gravity (MARG) sensors to compute the orientation, velocity, and position of the human lower limbs. Experimental results demonstrate the main benefit of using multiple sensors and sensor fusion algorithms, and that is the ability to handle drift errors that typically emerge after several minutes of operation. The authors identified augmented and virtual reality, rehabilitation, emergency responders, etc. as potential applications for their solution.

The previous authors used the zero-velocity update (ZUPT) method to handle measurement noises in the cyclical nature of human walking, i.e., the estimated walking speed when the foot is flat on the ground is zero. Brzostowski [22] researched how the human walking speed can be estimated with a novel method based on transient artifact reduction algorithm (TARA) to estimate the low-frequency updates in the gate signal. The method relies on the fact that the drift in the estimated walking velocity is mainly comprised of low-frequency components. To remove these components, the method applies nonlinear signal processing and sparse modeling. The main advantage of this approach is that additional infrastructure or sensors are not needed to mitigate long-term drift.

For the remainder of this section, we will focus on those related works that investigate the elicitation of age and gender from gait information using image and inertial sensors.

### 2.2. Age and Gender Analysis

Early work by Gabell et al. [23] already identified that age has an effect on gait, identifying relevant temporal and spatial parameters, including a.o. stride time and step length. Similar gait analysis research was carried out in [24]. Follow-up research also explored the significance of gender on gait [25].

Riaz et al. [26] demonstrated it is feasible to elicit gender, age and height features from a single inertial sensor. Tested on 26 subjects and with the inertial sensor attached at four locations (chest, lower back, right wrist and left ankle), they collected 6D data (including accelerations and angular velocities across all axes) and segmented them into individual walking steps. Their classification rates in a standard 10-fold cross-validation experiment were up to 92.57% for gender prediction and up to 88.82% for age prediction when using the chest sensor. However, in a strict subject-wise cross-validation, the  results were down 6% to 20% compared to the standard 10-fold cross-validation, and this depending on the location of the sensor and the soft biometric being tested.

Lu et al. [27,28] explored the estimation of age and gender based on gait information. Contrary to our approach, they rely on images rather than inertial sensors. More recent work by Zhang et al. [29,30,31] also explored the elicitation of age and gender from image-based gait information. They propose, amongst others, a convolutional neural network (CNN)-based method for age group and gender estimation that leverages residual networks of residual networks (RoR). Depending on the datasets used, they achieve about 65% accuracy for age (about 95% when allowed to have a 1-off prediction) and a 93% accuracy for gender.

Follow-up work by Riaz on age predicting based on gait information was presented in [32]. They used inertial data collected from a sensor fixed at the chest during a normal human walk, from which they computed 50 spatio-spectral features for 86 subjects. The subjects were asked to walk straight on a 10 m flat surface, turn around, walk back to the starting point, and repeat this process a second time. The researchers explored different machine learning classifiers—including random forests, support vector machines, and multi-layer perceptrons—to estimate the age of the subjects. With a random forest regressor, the  best results they achieved was a 3.32 year average root mean square error (RMSE) and a 1.75 year mean absolute error (MAE) under a tenfold cross-validation and an average RMSE of 8.22 years under subject-wise cross-validation. To get meaningful results, the same subject should not occur both in the training and test set, which is not guaranteed in a traditional k-fold cross-validation with multiple gait traces per subject. This explains why the subject-wise cross-validation leads to worse results.

### 2.3. Contribution beyond the State-of-the-Art

In this work, we are not aiming to enhance the analysis of human motion activities, but rather to improve the accuracy of age and gender information elicited from IMU sensors. Although previous works explored this topic, studies carried out on a large population were scarce. Furthermore, some of the above works rely on reasonably long gait traces or assume the orientation of the sensor to be known or fixed. In this work, we do not assume these conditions to be true. We therefore report on the results we obtained for a larger population using short gait traces of only a few seconds, under circumstances where the orientation of the inertial sensor is not known upfront.

## 3. Gait Dataset and Features

In a gait system based on accelerometer and gyroscope sensory data, both linear acceleration including gravity $a\left(n\right)=\left(a_{x}\left(n\right),a_{y}\left(n\right),a_{z}\left(n\right)\right)$ and gyroscopic velocity $\omega \left(n\right)=\left(\omega_{x}\left(n\right),\omega_{y}\left(n\right),\omega_{z}\left(n\right)\right)$ signals are provided by the sensors at a given sample rate $f_{s}$.

### 3.1. The OU-ISIR Dataset Gait Action Dataset

The work is evaluated on the OU-ISIR labeled gait action dataset [33]. This dataset consists of 495 subjects with an almost equal gender distribution. The age of the test subjects ranges from 8 to 78 years old. Subjects wore a belt with 3 IMU sensors: left hip, right hip and back. 3D angular velocity $\omega \left(n\right)=\left(\omega_{x}\left(n\right),\omega_{y}\left(n\right),\omega_{z}\left(n\right)\right)$ and 3D acceleration $a\left(n\right)=\left(a_{x}\left(n\right),a_{y}\left(n\right),a_{z}\left(n\right)\right)$ are collected at a sampling rate of 100 Hz. Each subject performs two level walking sequences, one sequence where a slope was walked up and one down, one sequence where stairs were climbed and one descended. Relative location and orientation of the sensors between subjects and between sequences are invariant.

The biggest disadvantage is the constrained lab conditions during data collection. Yet, the OU-ISIR database is the largest inertial gait database w.r.t. the amount of subjects. Furthermore, all age groups are well represented and the gender population is well balanced.

### 3.2. Data Preprocessing

The collected data will be different depending on sensor location and orientation. It is possible to represent the data in a sensor-invariant way. However, sensor data from two entirely different body areas, e.g., foot versus hip, are fundamentally different. Small sensor displacements have shown to have an impact [34]. It is important to have training data sampled from the same underlying distribution as the data at test time.

Before the data from the inertial sensor is used for prediction, we describe the different methods we used to optionally transform the raw data into a more meaningful or understandable format.

#### 3.2.1. Raw Gait Sequence Data

The first approach we considered was to use the raw data directly, i.e., without any high-level feature extraction. The file format of the gait traces provided by the OU-ISIR dataset (http://www.am.sanken.osaka-u.ac.jp/BiometricDB/SimilarActionsInertialDB.html) we used for training and testing, includes the 3D gyroscope values (Gx, Gy, Gz), the 3D accelerometer values (Ax, Ay, Az), and the label for the inertial signal, as depicted below:

LineWidth:      7

Gx          Gy           Gz          Ax     Ay     Az    Label

-0.0625119      -0.169587       -0.273871       -0.09   -0.77   0.382   -1

-0.0571859      -0.169587       -0.263218       0.078   -0.928   0.056   -1

-0.0358809      -0.116324       -0.225934       0.098   -0.924   0.056   -1

...

The raw data was provided by the GAG2019 competition organizers [35] and collected at 100 Hz by 3 IMUZ sensors from ZMP Inc., Tokyo, Japan (https://www.zmp.co.jp/products/imu-z), which were located at the back waist of a subject and at the left and right waist. These sensors have a triaxial accelerometer and a triaxial gyroscope, and their dynamic ranges were respectively set at ±4 g and ±500 deg/s for capturing the human gait signal.

We feed the raw sequence data to our machine learning models. This allows us to analyze the impact of the data preprocessing step in the overall machine learning pipeline used for age and gender prediction.

#### 3.2.2. Vertical and Horizontal Acceleration Components

A rotation invariant representation of the data was extracted by the vertical and horizontal acceleration components derived from the accelerometer data as described by Lu et al. [36]. The vertical component was derived by approximating the direction of gravity as the mean of a(n) over a time frame with length *T*:

(1) $$G=\frac{\sum_{n=j}^{T+j}\left(a_{x}\left(n\right),a_{y}\left(n\right),a_{z}\left(n\right)\right)}{T}.$$

Then the vertical component of sample a(n) with n∈[j;j+T] is the dot product with the gravity G:

(2) $$a_{v}\left(n\right)=a\left(n\right)•G.$$

The horizontal component is defined as:

(3) $$a_{h}\left(n\right)=|a\left(n\right)-v\left(n\right)\frac{G}{|G|}|.$$

The norm of the horizontal vector $a_{h}\left(n\right)$ is taken, as its direction is meaningless. The gravity is approximated by taking the mean over a time frame of length *T*. The corresponding velocities $v_{v}\left(n\right)$, $v_{h}\left(n\right)$ are computed by integration, and their corresponding jerks $j_{v}\left(n\right)$, $j_{h}\left(n\right)$ are computed by differentiation.

This leaves us with 6 new components for every sample *n*. This procedure is repeated for a sliding window of length *T* and step size T/2.

#### 3.2.3. Gait Dynamics Image

Gait dynamics image (GDI) was first proposed by Zhong and Deng [37]. They represented linear acceleration and angular velocity in a orientation invariant manner by taking the cosine similarity with a reference vector. This similarity was calculated separately for angular velocity and acceleration as follows:

(4) $$S_{\omega }\left(t_{1},t_{2}\right)=\frac{\omega \left(t_{1}\right)•\omega \left(t_{2}\right)}{|\omega \left(t_{1}\right)||\omega \left(t_{2}\right)|}$$

(5) $$S_{a}\left(t_{1},t_{2}\right)=\frac{a\left(t_{1}\right)•a\left(t_{2}\right)}{|a\left(t_{1}\right)||a\left(t_{2}\right)|}.$$

The reference vector was iteratively renewed. The amount of similarities calculated for a single reference vector is the time delay *m*. The time delay captures the local context, i.e., the motion interactions between the reference vector and its successors. The GDI is defined as:

(6) GDI(i,j)=S(j,i+j−1)withi=0,⋯,m−1andj=0,⋯,N−m,

where *N* is the length of the sequence. *S* is either $S_{a}$ or $S_{\omega }$, which respectively results in a GDI for acceleration and angular velocity.

#### 3.2.4. Angle Embedded Gait Dynamics Image

Angle embedded GDI (AE-GDI) [38] is an extension of GDI that is invariant to scaling and translation, by relying on the angles between the vectors in 3D space. Furthermore, the full gait trace is split up w.r.t. starting positions that are found in an alignment procedure. While there is a multifold of alignment procedures proposed in literature, we followed the one as described by Zhao and Zhou [38]. We extracted windows of fixed length *N*. The windows start at the starting positions found in the alignment step. The similarity function is defined as:

(7) $$\begin{array}{c} AEGDI_{v}\left(i,j-m\right)=\arccos\left(\frac{\left(v(j-i)-v(j))•(v(j+i)-v(j)\right)}{|(v(j-i)-f(j))||(v(j+i)-v(j))|}\right)\\ \mathrm{with}\;i=1,\cdots ,m\;\mathrm{and}\;j=m,\cdots ,N-m-1, \end{array}$$

with *m* the chosen time delay, m∈(0,N/2), *v* is either the acceleration vector a or the angular velocity vector ω.

### 3.3. Automated Feature Extraction with Deep Learning

**Figure 1:**
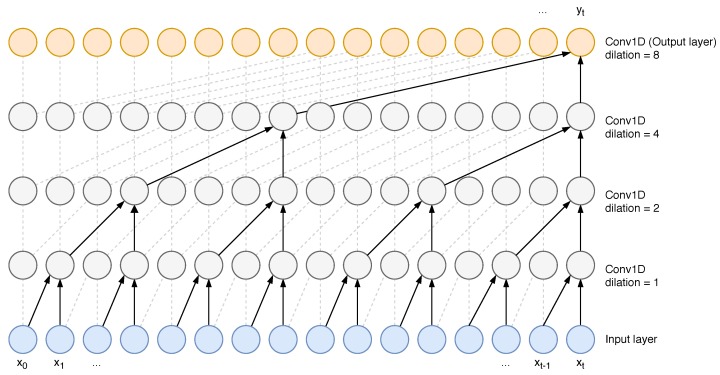
Causal, dilated convolution with kernel size 2 and dilation factors, 1, 2, 4 and 8. Figure adapted from [40].

Deep learning allows to obtain high-level representations of the input space. Two dimensional CNNs have proven to be very successful in the visual domain, i.e., representing images. We look for a high-level representation of the gait sequences by means of temporal convolutional networks (TCNs) as proposed by Bai et al. [39]. When dealing with time series, TCNs contribution is two-fold: to map variable-length input sequences to outputs of the same length, and to constrain an output at time *t* to depend only on inputs that have been observed in the past, within the interval [0,t]. The first goal is achieved by using a zero-padding trick that enforces the input-output size matching. TCNs use causal convolutions for convolving the output at time *t* with past input values, up to a specific time instant. This time instant is linked to the receptive field of TCNs kernels, that only grows linearly w.r.t. the network depth. This limitation is overcome with dilated convolutions, firstly introduced by Oord et al. [40] and represented graphically in Figure 1. They allow for exponential growth of the receptive field, or history of values, by introducing a dilation factor as in

(8) $$F\left(s\right)=\sum_{i=0}^{k-1}f\left(i\right)*x_{s-d*i},$$

where *d* is the dilation factor, *f* is the filter and *k* is the filter size. When d=1 we have a one-dimensional convolution, otherwise a fixed step between inputs of the previous layer is introduced. Following the authors’ suggestion, when multiple dilated layers are stacked, *d* is set to grow exponentially. This accounts for low-level spatial accuracy as well as a global view of our input because of a large receptive field, while the overall complexity is kept low with a number of parameters that only grows linearly with the network depth.

Different layers were also involved in the extractor architecture. TCNs layers were intertwined with dropout layers: randomly-chosen channels were zeroed-out during the training phase and re-activated in testing for helping with regularizing the network. Following best practices, L2 was also taken into account as a regularization tool. Moreover, a global average layer was used to flatten the output of the extractor. We represent the variables in the multivariate time series as channels.

The extracted features are conveyed in a sequence of dense layers. The work of these units is to learn meaningful patterns for our target problems. In both cases, the network was trained to optimize a goal function: mean absolute error is minimized in the age case while classification accuracy was maximized for gender. This was achieved by minimizing two loss functions that approximate our nominal targets: mean absolute error (cfr. Equation (9)) for age and binary cross-entropy (cfr. Equation (10)) for gender.

(9) $$MAE\left(l,p\right)=\frac{1}{N}\sum_{i=0}^{N-1}|p_{i}-l_{i}|$$

(10) H(l,p)=(l∗log(p)+(1−l)log(1−p)).

### 3.4. Experimental Protocol

The dataset was split in a training $U_{tr}$ and testing $U_{te}$ set w.r.t. subjects, such that $U_{tr}\cap U_{te}=\emptyset$. Special care was taken to retain age and gender distributions. To tune model parameters, the training $U_{tr}$ set needs to be further split in a training $U_{tr}^{\prime }$ and validation $U_{val}^{\prime }$ set. With $U_{tr}=U_{tr}^{\prime }\cup U_{val}^{\prime }$ and $U_{tr}^{\prime }\cap U_{val}^{\prime }=\emptyset$. Depending on the size of the search space, a holdout or k-fold cross-validation procedure is used to select the validation set. A holdout procedure has the obvious disadvantage of being more biased to the validation set. While k-fold cross-validation averages the results over different runs, it increases computation time by a factor k. Once the ideal model parameters were found, using $U_{tr}^{\prime }$ for training and $U_{val}^{\prime }$ for validation, the model was retrained with the full training set $U_{tr}$.

The classifiers for gender are compared in terms of accuracy, precision, recall and F1-score. We encoded male as 1 and female as 0. A false positive fp occurs when male is predicted while the correct label was female. Vice versa, predicting female for a male instance, is called a false negative fn. True positive tp and true negative tn occur when respectively male and female are correctly predicted.

(11) $$accuracy=\frac{tp+tn}{tp+tn+fp+fn}$$

(12) $$precision=\frac{tp}{tp+fp}$$

(13) $$recall=\frac{tp}{tp+fn}$$

(14) $$F_{1}=2*\frac{precision*recall}{precision+recall}.$$

The regressors for age were compared in terms of mean absolute error MAE.

(15) $$MAE=\frac{\sum_{i=1}^{n}|x_{i}-y_{i}|}{n},$$

where $x_{i}$ is the predicted age for instance *i* and $y_{i}$ is the ground truth. *n* is the total amount of samples, i.e., the amount of samples in test or validation set. Mean squared error will punish big mistakes, while mean absolute error weighs all mistakes equally, which makes it more interpretable.

Note that the data splits were made w.r.t. subjects. Every subject performed multiple walks under varying conditions, i.e., slope up/down, level walk, stairs up/down. Predictions can thus be provided for each action type, or they can be averaged, to provide a single prediction based on all the acquired data. Furthermore, some methods further segmented the different walks. If that was the case, the predictions for the different segments were always averaged over either the specific activity or all activities.

## 4. Machine Learning Pipelines for Age and Gender Prediction

We built multiple systems with the building blocks described in Section 3. We describe the pipelines for every system. They differ in data preprocessing, feature extraction and classification and regression algorithms.

### 4.1. Automated Selection of Learning Algorithm and Hyperparameters

This system automated gender and age prediction using AutoWeka 2.0 [41] as the underlying software framework. We relied on 100+ high-level features derived from the accelerometer traces [9], i.e., the gyroscope values are excluded in the feature engineering phase. The feature engineering phase aggregated the raw samples in intervals ranging from 2 to 20 s, and we analyzed the results by relying on different combinations of input data:

- Sensor position: all sensors vs. left sensor vs. right sensor vs. center sensor
- Activity: all activities vs. only walking activity.

The AutoWeka framework explored different traditional machine learning algorithms (i.e., no deep learning) and different parameters for these algorithms. To compare for the best results, AutoWeka was allowed up to 1 h of computation time and 8 GB of memory to compute the best model. To do so, it internally uses 10-fold cross validation on the training set (80% of the users). We evaluated the test set (20% of the users) on these different models to analyze their generalization capabilities for unseen users.

The outcome for the best machine learning (ML) algorithm depended on the above experimental combinations, and included instances of the following algorithms for age:

- weka.classifiers.trees.RandomForest
- weka.classifiers.functions.SMOreg
- weka.classifiers.meta.RandomSubSpace
- weka.classifiers.lazy.Kstar

The experiments for gender resulted in the following algorithms performing the best for one of the above combinations:

- weka.classifiers.trees.RandomForest
- weka.classifiers.functions.SMO
- weka.classifiers.meta.AttributeSelectedClassifier
- weka.classifiers.lazy.Ibk
- weka.classifiers.meta.RandomSubSpace

The results obtained through AutoWeka are not guaranteed to offer the best outcome. Better results are achieved when AutoWeka is given more time, CPU and memory resources to find the best performing algorithm and hyperparameters. For the competition test dataset, we used 1 model to compute the age values, and another model to compute the gender values.

### 4.2. Hidden Markov Model and Universal Background Model

**Figure 2:**
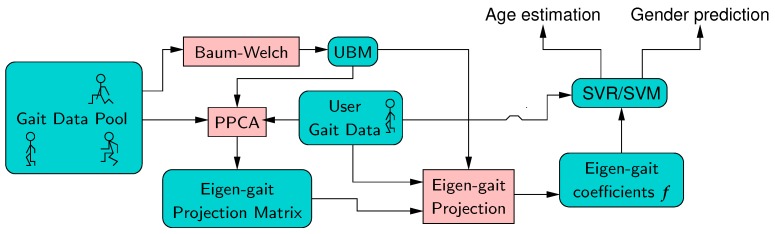
Block diagram of the proposed system.

This system models signatures using hidden Markov models (HMMs). Each model contains S=8 states, with a Gaussian mixture model (GMM) output probability density function. During the system offline training, an universal background model (UBM) is trained using the Baum-Welch recursion on gait signals from a population. Then, an eigen-gait projection matrix is derived using the probabilistic principal components analysis explained in [42] for GMMs and extended in [43] for HMMs. This matrix is usually called the total variation matrix in the i-vector framework. Sequences from a given user are then projected using the UBM and the total variation matrix into a set of eigen-features, which are used as a fixed length model of the user. In the system we delivered, E=30 eigen-coefficients per state are derived. A support vector regressor (SVR) is derived from eigen-gait coefficients from a population with labeled age, and a support vector machine is derived from eigen-gait coefficients from a population with labeled gender. These are used to estimate the age or gender respectively from the eigen-gait coefficients obtained for any test population. These processes are illustrated in Figure 2.

In this system, gait sequences are represented by rotation invariant features called gait dynamic images (GDIs) [37]. A GDI is extracted for the accelerometer, and another from the gyroscope. First differences of these GDIs are also computed. For each of these signals, Short Time Fourier Transform is used to extract a frequency representation.

### 4.3. Deep Learning with Temporal Convolutional Networks and Dense Layers

Recent trends underline the effectiveness of deep networks in modelling time series as opposed to traditional ML techniques. These networks can reduce highly complex problems, such as feature extraction, to the fairly easier task of optimizing a single model. Given the nature of the input data for the GAG competition, i.e., human gait, it is clear how this solution applies in our context. In the following, we present our architecture to solve both gender classification and age estimation problems.

We can split our model into two parts: a feature extractor, which is a temporal convolutional network (TCN), and a number of fully connected layers, or dense layers. The latter is stacked to the former and the architecture is terminated by a task-specific output layer. As stated above, this architecture applies for both tasks. However, optimization is performed, according to the use case, during training.

As proposed by Bai et al. [39], we apply temporal convolutional networks (TCNs) as a feature extractor. TCNs convolve input sequences in a dilated causal fashion. In causal convolutional layers, several filters slide over the input covering their receptive field, which represents the history of values up to a specific time *t*. Given that the receptive field, or the history of values, can only grow linearly with the depth of our network, this type of architecture would limit our glance on the sequence. For this reason, a dilatation factor was applied that increases the receptive field while keeping the complexity of the architecture low. This allows for local accuracy and a global view at the same time. In our model, we exploit the ability of such networks to capture relevant features from the input space as well as their capacity of handling variable-length input sequences.

**Figure 3:**
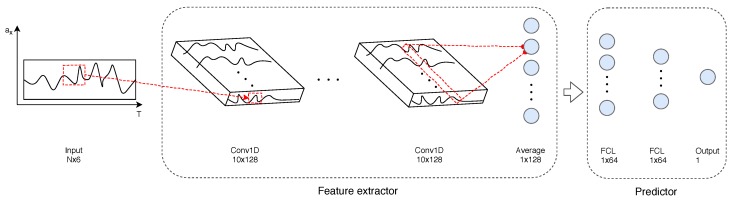
Network architecture.

The extracted features are then conveyed in a sequence of dense layers. The work of these units is to learn meaningful patterns for our target problems. In both cases, the network is trained to optimize a goal function: mean absolute error is minimized in the age case while classification accuracy is maximized for gender. This is achieved by minimizing two loss functions that approximate our nominal targets. The whole architecture is shown in Figure 3. This is a simplified version of the pipeline which takes into account the processing a sequence of measurements of one sensor in a fixed direction, i.e., $a_{x}$, while our input data has six channels given by the output of the triaxial accelerometer and gyroscope of the IMUZ sensor. The time series is fed to the causal convolutional layers which produce a number of vectors equal to the number of chosen filters, i.e., 128 in our case. The feature extractor was finished by a layer which averages every one of our output vectors with size dictated by the kernel size of the filter, i.e., 10 in our example. The latter is important to preserve the time dependency of the input while providing us with a one-dimensional vector of size 128 to be used as the predictor input. The pre-processing of the input sequence is left out of the scheme.

Our aim is to achieve an effective representation of the input space. To this extent, we provide the network with the raw input sequences of different sensors, including different activities but pruning away invalid ones, i.e., −1 labels. Two filtering steps were also carried out: first, we deleted sequences belonging to users who where absent from the labelled list of subjects provided by the competition organizers; secondly, we excluded sequences shorter than a specific period. At the end of the training procedure, we retained the best model w.r.t. to the validation accuracy.

**Figure 4:**
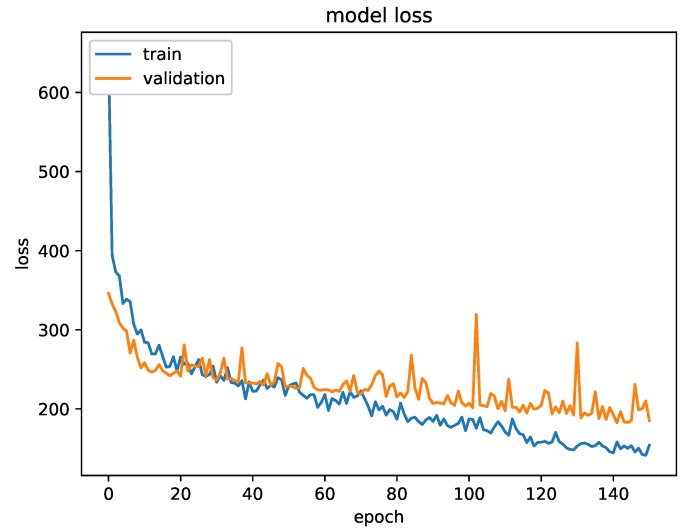
Train and validation mean absolute error (MAE) during training in the regression case.

The models underwent a phase of hyper-parameters optimization including activation layers, number of layers and several regularization techniques (e.g., droupout layers, Gaussian noise, kernel regularization etc.). This led to a pair of models that are similar in architecture yet profoundly different in the chosen parameters. Figure 4 shows the effectiveness of regularizing our model while training: the validation accuracy is kept limited throughout the whole process.

The only predetermined difference, intrinsic to the nature of the problem, is the activation function of the output level. We selected a single, fully-connected layer with two variants: a linear function, which produces a continuous output, has been chosen for the regression problem, i.e., the estimation of age; while different non-linear activation functions have been considered when dealing with the binary classification problem, i.e., the estimation of gender.

**Figure 5:**
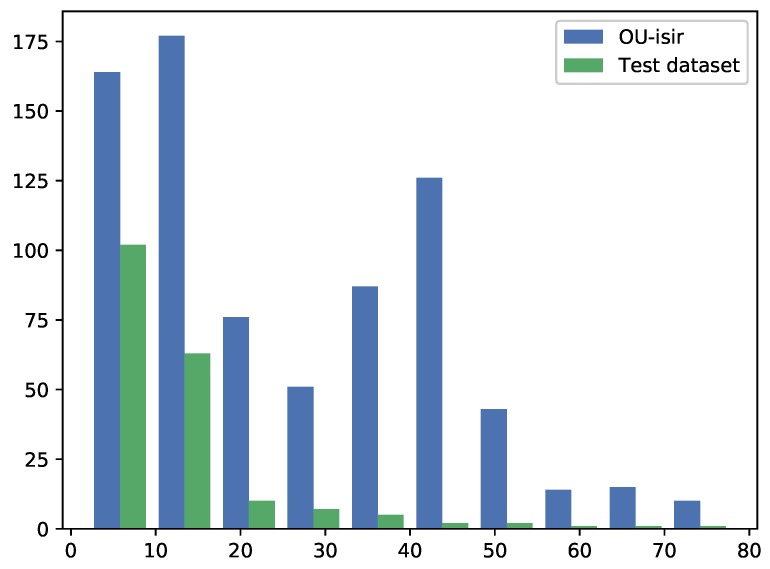
Comparison between labelled and GAG-2019 competition test datasets distributions in the regression case.

The GAG-2019 competition test sequences were fed into the finalized model as they are, that is no pre-processing has been carried out. Figure 5 shows the distribution of the age model output for train and GAG-2019 competition test sequences.

The following method follows a similar deep learning approach but with an orientation independent representation of the gait sequences.

### 4.4. Deep Learning with Orientation Independent Representation

The sensor position and orientation are unknown in the competition test dataset. A correction for sensor position is hard, as a sensor on a different body part will measure fundamentally different data. However, we can take an orientation-independent approach. Vertical and horizontal acceleration components were derived as described in Section 3.2.2. This representation was inspired by the work from Lu et al. [36]. Their corresponding velocities $v_{v}\left(n\right)$, $v_{h}\left(n\right)$ were computed by integrating the linear accelerations, and their corresponding jerks $j_{v}\left(n\right)$, $j_{h}\left(n\right)$ are computed by differentiation. The gyroscope data was not used.

The sequences within the unlabeled competition dataset were relatively long. Therefore we decided to send in two solutions obtained from this network. (1) One where one prediction was done for the whole sequence, and (2) one where the sequence was split in arbitrary chunks. The final prediction is the mean of the individual chuck predictions.

### 4.5. Deep Learning with Orientation Invariant Representation of Gait Based on GDIs

We first performed a preprocessing step, where Angle Embedded Gate Dynamic Images (AE-GDIs) [38] are extracted. AE-GDIs are an orientation invariant representation of gait based on GDIs as proposed by Zhong and Deng [37]. These AE-GDIs were fed to the deep neural network. We can split our model into two parts: a feature extractor, which consists of 2D convolutions and pooling layers, and a number of fully connected layers, or dense layers. The latter is stacked to the former and the architecture is terminated by a task-specific output layer. As stated above, this architecture applies for both tasks. However, optimization was performed, according to the use case, during training.

This network was inspired by Zhao and Zhou [38]. They compare TCNs, the architecture used in the other deep learning approaches, and normal 2D convolutions. They conclude that GDIs and AE-GDIs are more like images and therefore thrive with architectures from computer vision, i.e., stacked 2D convolutions.

## 5. Evaluation

The above models were applied on the test dataset provided by the competition organizers. As outlined at http://www.am.sanken.osaka-u.ac.jp/GAG2019/, this test dataset contained 50% of gait traces collected in a similar setting as those of the OU-ISIR datasets. The other half were collected in the wild on an almost flat ground. Furthermore, to make the competition more challenging, sensors were fixed in a backpack while their orientations were unknown to the competition participants.

### 5.1. Baseline Comparison on the Same Dataset

A systematic comparison with results in the related work is not trivial, due to differences in the number of test subjects, different characteristics with the subjects themselves, the length of their gait traces, the equipment used to collect the data, the circumstances in which the data was collected, the way errors are reported (e.g., traditional vs. subject-wise cross-validation) and the lack of information to reliably reimplement previous work. This section therefore reports on the independent evaluation by the GAG2019 competition organizers and the comparison of our results with those of the other competing teams.

From the 18 teams registered, only 10 teams submitted results for the competition. Participants were allowed to submit multiple solutions for the age and gender prediction. The best algorithms for each team are depicted in Table 1. These evaluation results were provided by the competition organizers. For more details we refer to [17]. The prediction results of our team are given under team identifier GAG2019121202. As the table shows, we achieved the lowest error rates for both the age and gender competition.

Table 2 shows our results for the different methods. It is clear that the orientation independent AE-GDI representation combined with the deep learning method achieved the best results. The high error rate for the HMM method in the gender category can be explained by the fact that in some test cases, the model could not make a reliable prediction. Rather than random guessing the gender, we purposefully decided to not provide a prediction. As a consequence, an empty prediction was also considered erroneous. If we would have randomly picked male or female for the gender prediction, the error rate of the empty predictions would have dropped with about 50%. This clarifies the high error rate, and explains why having the HMM predict the opposite gender would not have resulted in a 41.7526% error rate.

Additionally, the last row depicts the predictions based on a simple voting (gender) or averaging (age) of the previous methods. Specifically for the age prediction, it seems to outperform many of the individual methods. Also, automated model selection and hyperparameter tuning method stood its ground for age prediction, also when comparing with the best results of the other competition participants.

### 5.2. Discussion and Implications

Compared to the state-of-the-art, we were able to improve the accuracy of the predictions on even larger populations and with shorter gait traces. Key to the success of our methods is the ability to represent gait traces in a sensor orientation independent manner to be robust against a wide variety sensor placements where the position on the body remains near invariant. This way, we can handle changing sensor orientations during the training and test phases. Contrary to the related works on gait-based motion analysis, long-term drift errors were not a significant concern due to the relatively short gait traces we used in our experiments.

The error rates provided in Table 2 are in fact an overestimation for some of the tested methods. The reason for this overestimation is that for certain methods, such as the HMM approach, no predictions were made for several test subjects whenever a manual analysis of the raw data indicated that a gait pattern could not be reliably identified. Rather than simply proposing a random value (which would give a 50% chance of being correct for the gender prediction), we offered no value which was considered as incorrect by default, hence increasing the error rate for that particular method.

Our work shows that even a limited amount of information may reveal sensitive personal information of a subject. While not 100% accurate, the predictions are close enough to be valuable for applications such as recommender systems and targeted advertising. The fact that accelerometer information is available to websites through standardized browser APIs [44], makes the leakage of age and gender information a non-negligible privacy concern. Previous research has shown that sensitive information such as gender can be leaked, for example, by ratings provided by users. As a result, complementary countermeasures such as BlurMe [45] were proposed to disguise this sensitive data while achieving an insignificant effect on the recommendations provided to that user. However, similar countermeasures are far less trivial to implement for gait traces. Given the growing availability of gait data and the ease with which the data can be collected, one may reasonably expect that the accuracy of age and gender prediction models will further increase in the future. These observations make the privacy concern even more distressing.

## 6. Conclusions

In this work, we reported on the results we obtained in the OU-ISIR Wearable Sensor-based Gait Challenge [17] where the objective was to predict age and gender on a short trace of accelerometer data. Our team achieved the best predictions in both competitions. While the results are impressive on their own, it demonstrates the need that sensitive personal information may be hidden in data where we the least expect it. Indeed, while activity recognition and security application may process accelerometer traces for benign purposes, it should be clear that subjects may unknowingly leak sensitive information about themselves. With the General Data Protection Regulation (GDPR) [46] being implemented since 25 May 2018, application developers and service providers must pay attention to the way they tackle private data collection and processing. While Article 25 emphasizes the data minimization principle, and only collect data useful for their own purposes, we have shown this is not feasible for these stakeholders to anticipate privacy concerns upfront.

As future work, we will investigate adequate measures to mitigate unintended information leakage such that sensors and other data streams can no longer be abused as a side channel for gathering sensitive information or private traits with the user’s consent.

## Figures and Tables

**Table 1:** Best gender and age prediction results for the 10 teams that submitted results, with our team results marked in bold.

Team	Gender (% of Mistakes)	Age (Mean Absolute Error)
GAG2019112901	45.8763	20.0670
GAG2019113001	38.6598	7.7824
GAG2019120402	31.4433	6.9278
GAG2019120601	47.9381	12.1340
GAG2019120701	30.4124	6.4381
GAG2019121201	30.9278	9.2107
**GAG2019121202**	**24.2268**	**5.3879**
GAG2019121501	24.7423	6.6175
GAG2019122501	30.9278	7.0499
GAG2019122601	50.0000	13.6237

**Table 2:** Gender and age prediction results for the different methods, with the best one marked in bold.

Method	Gender (% of Mistakes)	Age (Mean Absolute Error)
AutoWeka 2.0	41.7526	7.1959
HMM	58.2474	9.6186
TCN	39.6907	12.2990
TCN + Orientation Independent (1)	34.5361	8.1875
TCN + Orientation Independent (2)	32.9897	8.1942
**CNN + AE-GDI**	**24.2268**	**5.3879**
Ensemble	35.5670	5.9433
